# Increase in markers of airway inflammation after ozone exposure can be observed also in stable treated asthmatics with minimal functional response to ozone

**DOI:** 10.1186/1465-9921-11-5

**Published:** 2010-01-19

**Authors:** Barbara Vagaggini, Maria Laura E Bartoli, Silvana Cianchetti, Francesco Costa, Elena Bacci, Federico L Dente, Antonella Di Franco, Laura Malagrinò, Pierluigi Paggiaro

**Affiliations:** 1 Cardio-Thoracic and Vascular Department, University of Pisa, Pisa, Italy

## Abstract

**Background:**

The discrepancy between functional and inflammatory airway response to ozone has been reported in normal subjects, but few data are available for stable asthmatics regularly treated with inhaled corticosteroids.

**Methods:**

Twenty-three well controlled, regularly treated, mild-to-moderate asthmatic patients underwent two sequential randomised exposures to either filtered air or ozone (0.3 ppm for 2 hours) in a challenge chamber. Pulmonary function (PF) was monitored, and patients with FEV1 decrease greater than 10% from pre-challenge value were considered as responders. Immediately after each exposure, exhaled breath condensate (EBC) was collected to measure malondialdehyde (MDA). Six hours after each exposure, PF and EBC collection were repeated, and sputum was induced to measure inflammatory cell counts and soluble mediators (IL-8 and neutrophil elastase). The response to ozone was also evaluated according to the presence of polymorphism in oxidative stress related *NQO1 *and *GSTM1 *genes.

**Results:**

After ozone exposure, sputum neutrophils significantly increased in responders (n = 8), but not in nonresponders (n = 15). Other markers of neutrophil activation in sputum supernatant and MDA in EBC significantly increased in all patients, but only in nonresponders the increase was significant. In nonresponders, sputum eosinophils also significantly increased after ozone. There was a positive correlation between ozone-induced FEV1 fall and increase in sputum neutrophils. No difference in functional or inflammatory response to ozone was observed between subjects with or without the combination of *NQO1wt*- *GSTM1null *genotypes.

**Conclusions:**

Markers of neutrophilic inflammation and oxidative stress increase also in asthmatic subjects not responding to ozone. A greater functional response to ozone is associated with greater neutrophil airway recruitment in asthmatic subjects.

## Background

Ozone is a potent oxidant known to induce a variety of respiratory effects, including cough, increased airway reactivity, decrease in lung function and neutrophilic airway inflammation [1]. Recent evidence supports a role for environmental chronic exposure to ozone in the development of asthma and in triggering asthma attacks [2,3]. Ozone exposure imposes an oxidative burden on the lung both by directly oxidizing biomolecules, thereby generating reactive oxygen species (ROS), and by inducing inflammatory mediator production and release, with activation of inflammatory cells and further release of ROS [4]; this process causes acute and chronic airway damage, which results in bronchoconstriction and bronchial hyperresponsiveness [5].

Many studies have reported an increase in markers of neutrophilic activation in induced sputum or in bronchoalveolar lavage (BAL) after ozone exposure in healthy ad asthmatic subjects [6,7], as well as an increase in markers of oxidative stress in lung tissue and BAL fluid of animals exposed to high ozone concentrations [8]. There is, however, considerable interindividual variability in the magnitude of pulmonary response, both in terms of functional and inflammatory reaction to ozone exposure, with a large proportion of subjects showing no significant change in airway calibre after controlled experimental ozone exposure in laboratory [9]. No specific determinants of poor functional response to ozone have been demonstrated, apart from age and levels of exposure [10,11]. Most experimental studies in humans have reported no correlation between functional response and severity of inflammatory response measured in induced sputum or bronchoalveolar lavage fluid [12-14]. In particular, very few data have been reported in asthmatic subjects.

In humans, increased levels of malondialdehyde and 8-isoprostane have been measured in breath condensate after O3 exposure [15,16].

Polymorphism of two oxidative stress related enzymes, Glutathione-S-Transferase *M1 (GSTM1) *and NAD(P)H:quinone oxidoreductase (*NQO1*), has been associated with an increased susceptibility to ozone exposure injury. There are studies showing that the deleted form of Glutathione-S-Transferase M1 enzyme (*GSTM1 null*), resulting in a complete lack of its enzymatic function, associated with the pro187 form of NAD(P)H:quinone oxidoreductase (*NQO1*) enzyme, induces greater acute airway and inflammatory responses to ozone [17,18].

The aim of our study was to evaluate the effects of ozone exposure on functional and inflammatory airway responses in 23 mild-to-moderate, stable asthmatic subjects, regularly treated with inhaled corticosteroids. We and other authors have demonstrated that inhaled or oral corticosteroids can blunt neutrophilic airway inflammatory response to ozone in asthmatics [19-21], but have little effect on airway calibre. Therefore, we would like to verify whether, in asthmatic subjects regularly treated with inhaled corticosteroids, a dissociation between functional and inflammatory airway response to ozone can be observed.

## Subjects and methods

### Subjects

We selected 23 nonsmoking, mild-to-moderate stable asthmatic subjects, regularly treated with inhaled corticosteroids (median daily dose: 500 μg of BDP-equivalent, range: 200-1000), associated with long-acting beta2-agonists in 19 out of 23 subjects, all aged under 50 years. Asthma severity and level of control were assessed according to International Guidelines [22]. All subjects were in stable phase of disease and had had no upper respiratory tract infection or acute asthma exacerbation in the last 4 weeks. Asthma treatment was withdrawn 24 hrs before each exposure. Main characteristics of examined subjects are reported in Table 1.

**Table 1:** Characteristics of the asthmatic subjects examined

	All subjects	Responders	Nonresponders
Number	23	8	15

Age, yrs (M ± SD)	32.6 ± 10.8	31.1 ± 13.2	33.4 ± 9.8

Sex (Male/Female)	13/10	6/2	7/8

Atopy (yes/no)	17/6	5/3	12/3

Smoking habit (yes/ex/no)	0/5/18	0/2/6	0/3/12

FEV1, % predicted (M ± SD)	96.9 ± 12.0	93.4 ± 15.9	98.8 ± 9.4

PD20FEV1 meth, mg (GM)	0.263	0.366	0.218

ICS, μg/d (median, range)	500(200-1000)	500(250-1000)	500(200-1000)

FEV1:Forced expiratory volume in one second ; M ± SD: mean ± standard deviation; GM: geometric mean. PD20FEV1 meth: cumulative dose of methacoline provoking a 20% decrease in FEV1 from baseline value; ICS: Inhaled corticosteroids.

### Study design

On two different days, at least 2 weeks apart, all subjects were randomly exposed to either ozone (0.3 ppm) or filtered air for 2 hours in a challenge chamber, while excercising on a cycloergometer. Before and after each exposure, they underwent pulmonary function test (PFT), collected exhaled breath condensate (EBC) and measured nitric oxide levels in exhaled air (eNO). Six hours after the end of the exposure, PFT, EBC collection, eNO measurements were repeated, and hypertonic saline (HS)-induced sputum was collected.

The study protocol was approved by the local University Ethic Committe, and an informed consent was obtained by each patient before entering in the study.

Subjects were divided in responders and nonresponders according to their responsiveness to ozone, corrected by the changes in airway calibre after exposure to air. Airway responsiveness to ozone was defined as the difference between FEV_1 _values (L) measured after O_3 _and after air exposure (ΔFEV_1 O3-Air_), according to the following formula:

Subjects with ΔFEV_1 O3-Air _greater than 10% were considered as "responders" [12].

Venous blood was taken before the first exposure, to evaluate the genotypic combination of NAD(P)H:quinone oxidoreductase (*NQO1*) and Glutathione-S-Transferase M1 (*GSTM1*) enzymes. Functional and inflammatory airway responses of subjects bearing both *NQO1wt *and *GSTM1null *genotypes were compared with those of the other genotypic combinations.

### Challenge chamber

All subjects were exposed to ozone for 120 min in a 9-m^3 ^static challenge chamber made of glass and aluminium [19], while exercising on a stationary cycloergometer at work load predetermined to produce a ventilation rate of 25 L/min/m^2 ^of body surface area for twenty min every hour. Ozone was generated by a corona discharge O_3_-generator (Rancon Instruments SpA, Milano, Italy) connected to a cylinder of purified air. Ozone output into the chamber was 0.5 L/min. An O_3_-analyser (Photometric O_3 _Analyser 400, Rancon Instruments SpA, Milano, Italy) connected to the chamber by a tubing circuit, continuously monitored gas concentration. Mean ozone concentration was maintained at about 0.3 ppm throughout the exposure. A fan in the chamber ensured adequate gas mixing and circulation.

### Sputum induction and processing

Hypertonic saline solution (NaCl 4.5%) was nebulized with an ultrasonic nebulizer (2.8 ml/min output; Sirius, Technomed, Firenze, Italy) and inhaled for three 5-minute periods, for up to 15 min. Every 5 min after the start of nebulization, patients were asked to carefully rinse their mouth and throat in order to discard saliva and to try to cough sputum into a clean container; FEV1 was then measured. Nebulization was stopped after 15 min or when FEV1 fell by 20% or more from baseline values.

Sputum samples were processed within 2 h from collection, and more viscid and denser portions were selected and processed as previously described [23]. Briefly, samples were homogenized by adding 0.1% dithiothreitol in a shaking bath at 37° C for 15 min and centrifuged to separate cells from supernatant. Supernatant was stored at -80°C for further analysis. The cell pellet was resuspended in phosphate-buffered saline for viability test and total cell count; aliquots were cytocentrifuged (Cytospin; Shandon Scientific, Sewickley, PA, USA) to prepare slides for differential cell counts. At least 300 inflammatory cells were counted. Macrophage, lymphocyte, neutrophil, eosinophil values were expressed as percent of total inflammatory cells.

Slides with cell viability < 50% or with an amount of squamous cells such that 300 inflammatory cells could not be counted were considered inadequate and discarded. Our reproducibility for sputum inflammatory cell counts was previously assessed and excluding lymphocytes (RI: 0.23), was considered as satisfactory: RI was 0.90 for macrophages, 0.88 for neutrophils, 0.82 for eosinophils [24].

### Exhaled breath condensate (EBC) collection

EBC was collected by cooling exhaled air with a specifically designed condenser (Ecoscreen, Jaeger, Wurzburg, Germany). Subjects breathed tidally for 15 min through a two-way non-rebreathing valve in order to prevent inspiratory and espiratory air mixing and saliva trapping [25]. The condensate thus obtained was immediately stored at -30°C for further analysis.

### Exhaled Nitric Oxide (eNO) measurement

NO was measured in exhaled air using a Nitric Oxide Analyzer (Sievers NOA 280, Boulder, CO, USA). Under visual feedback, patients performed a single slow exhalation (30-45 sec) from total lung capacity through a resistance, keeping a constant expiratory flow of about 50 L/min; eNO concentration at mouth level was recorded throughout expiration. At least three acceptable manoeuvres with eNO variability lower than 10% were obtained, and the mean value was considered.

### Biochemical analysis

#### Cytokines and neutrophilic biomarkers

Sputum supernatant IL-8 levels were measured with a commercially available enzyme immunoassay (Euroclone, Milano, Italy) according to manufacturer's recommended protocol. The detection limit of the assay was 0.0625 ng/ml. The percentage of recovery, evaluated in 10 different samples, was 89.4%. Intra- and inter-assay coefficients of variation were 4.7% and 7.5%, respectively [26].

Sputum Neutrophilic Elastase activity (NE) in induced sputum supernatant was measured spectrophotometrically using the synthetic substrate methoxysuccinyl-ala-ala-pro-val-paranitroanilide (MeOSAAPVpNa) (Sigma ALDRICH Company Ltd. Poole, Dorset, UK). Activity was measured by assessing the change in absorbance at 410 nm on a microplate reader, and quantified by extrapolation from a standard curve of pure NE. The detection limit of the method was 6 ng/ml. Intra- and inter-assay coefficients of variation were 5.6% and 12% respectively. NE recovery, measured in 28 samples, was greater than 80% [26].

#### Oxidative stress biomarkers

Malondialdehyde (MDA) concentrations were measured in sputum supernatant and EBC samples according to the method described by Larstad et al [27]. Briefly, samples were derivatised with tiobarbituric acid and then measured by means of High Performance Liquid Chromatography with fluorescence detector (HPLC; Binary HPLC pump 1525 and 2475 multi λ fluorescence detector, Waters, Milano, Italy), using excitation and emission wavelenghts of 532 and 553nm respectively. Our detection limit was 0.006 μm/L, the intra- and inter-assay reproducibility were 0.9 and 10.4% respectively and the recovery 96%.

#### Genotypic characterization

*GSTM1 *and *NQO1 *genotypes were characterized by molecular biology techniques, using genomic DNA extracted with Nucleon BACC2 (Amersham International plc, Little Chalfont, Buckinghamshire, UK) from peripheral blood after buffy-coat enrichment, according to the procedures described elsewhere [17].

### Statistical analysis

Functional data are expressed as mean ± standard deviation, and compared between groups using unpaired Student's t-test. Comparison between repeated measurements in the same group was performed using ANOVA test. Inflammatory markers are expressed as median and range, and compared between groups using Mann-Whitney U test. The correlation between FEV_1 _response to ozone and O_3_-air changes in inflammatory markers has been evaluated using Spearman's rank correlation coefficient. A p value lower than 0.05 has been considered as significant.

## Results

Eight (34.7%) of the 23 subjects studied were defined as responders to ozone. No difference was found between responders and nonresponders for the main baseline characteristics (Table 1). Six subjects (26%) had the *NQO1w*t e *GSTM1null *genotypic combination.

### Functional response

Mean values of FEV_1_, FVC and VC at different time-points during exposures to air or ozone, divided into responders and nonresponders, are reported in Table 2. No difference in baseline values (expressed either as percentage or as absolute value) was found between ozone and air challenges in both groups. As expected, in addition to FEV_1 _decrease, responders also showed significant decrease in FVC and VC at the end of ozone exposure in comparison with pre-ozone exposure values, with complete recovery by 6 h after exposure. However, nonresponders showed mild but significant reduction in FEV_1_, FVC and VC at the end of ozone exposure only when compared with after air exposure.

**Table 2:** FVC, VC and FEV1 measured at different time-points before and after exposure to air or ozone, in responders and nonresponders

	Responders (n = 8)		Nonresponders (n = 15)	
	**Air**	**Ozone**	**Air**	**Ozone**

FVC bas	4.65 ± 0.78	4.70 ± 0.63	4.69 ± 0,97	4.56 ± 1.00

FVC 2h	4.69 ± 0.75	4.38 ± 0.79§#	4.63 ± 0.93	4.52 ± 0.93§#

FVC 6h	4.69 ± 0.84	4.61 ± 0.79	4.67 ± 0.99	4.56 ± 1.00

				

VC bas	4.60 ± 0.79	4.69 ± 0.70	4.60 ± 0.99	4.55 ± 0.99

VC 2h	4.67 ± 0.75	4.43 ± 0.80§#	4.67 ± 0.96	4.49 ± 0.89

VC 6h	4.64 ± 0.83	4.56 ± 0.77	4.72 ± 0.99	4.54 ± 1.04§

				

FEV1 bas	3.59 ± 0.76	3.73 ± 0.62	3.55 ± 0.86	3.41 ± 0.88

FEV1 2h	3.79 ± 0.72	3.40 ± 0.57§#	3.60 ± 0.88	3.52 ± 0.81§

FEV1 6h	3.80 ± 0.75	3.59 ± 0.58	3.69 ± 0,96	3.50 ± 0.87§

FEV1:Forced expiratory volume in one second; FVC: Forced vital capacity; VC: Vital capacity. # p < 0.05 from baseline values; §: p < 0.05 from air at the same time point. Data are expressed as mean ± SD.

There was no difference in functional response to ozone challenge between subjects bearing *NQO1wt *and *GSTM1null *genotypes and subjects bearing different genotypic combinations (ΔFEV1% _O3-Air_: 3.75 ± 5.83 vs 1.54 ± 13.26 %, ns).

### Inflammatory response

Inflammatory findings of the study subjects, grouped in responders and nonresponders, are reported in Table 3. Two subjects did not collect adequate sputum samples or breath condensate in at least one occasion, and they were thus excluded from analysis.

**Table 3:** Inflammatory cells and soluble mediators in induced sputum after either air or ozone exposure, in subjects grouped according to functional response to ozone exposure

	Responders (n = 8)		Nonresponders (n = 13)	
	**Air**	**Ozone**	**Air**	**Ozone**

Infamm. cells/ml (10^6^)	2.6(1.5-5.7)	3.6(1.2-10.8)	2.1(0.2-6.8)	3.1(0.7-14.0)

Macrophages/ml (10^6^)	0.9(0.3-2.2)	1.4(0.3-3.2)	0.8(0.1-2.5)	0.9(0.4-10)

Lymphocytes/ml (10^6^)	0.03(0-0.3)	0.09(0-0.4)	0.02(0-0.1)	0.02(0-0.1)

Neutrophils/ml (10^6^)	0.7(0.2-2.0)	1.7(0.5-9.2)§	0.6(0-6.3)	0.8(0.1-7.0)

Eosinophils/ml (10^6^)	0.09(1-1.9)	0.02(0-0.6)	0.02(0-1.7)	0.4(0-1.6) #§

Macrophages (%)	40.4(8-89)	34.3(8-77)	51.1(6-85)	48.2(2-75)

Lymphocytes (%)	1.5(1-6)	1.4(0-14)	0.6(0-5)	0.8(0-5)

Neutrophils (%)	28.2(8-79)	55.4(23-90)§	41.0(0-93)	30.9(6-98)

Eosinophils (%)	2.8(0-55)	0.2(0-11)	0.3(0-63)	11.6(0-45)#§

NE (ng/mL)	0.7(0-6)	2.1(0-9)	1.2(0-5)	1.5(0-6.5)

NE rec.(%)	42(1-61)	59(34-83)	41(9-62)	60(33-78) ^

IL-8 (ng/mL)	22.9(10-44)	36.3(3-62)	11.8(6-72)	26.3(3-94) §

Inflamm.: inflammatory; NE: neutrophil elastase; NE rec: NE recovery; IL-8: interleukin 8. Data are expressed as median (range). §:p < 0.05 from air exposure; ^ p = 0.056 from air exposure; #:p < 0.05 from responders.

After ozone exposure, sputum neutrophils significantly increased in responders, but not in nonresponders. Sputum IL-8 and NE did not show any significant increase in responders (p = 0.13 and p = 0.15 respectively), while in nonresponders IL-8 and NE increased after ozone exposure, although the increase in NE was only close to the statistical significance.

When the difference between sputum neutrophil percentage after ozone and after air for each subject (Δ N%) was considered, a significantly higher value was observed in responders than in nonresponders (15.2 [-1.3, 64.5] vs 0.15 [-21,2, 52.5] %, p < 0.05).

Sputum eosinophils (both in absolute and percent value) after ozone exposure were significantly higher than after air exposure only in nonresponders.

**Figure 1:**
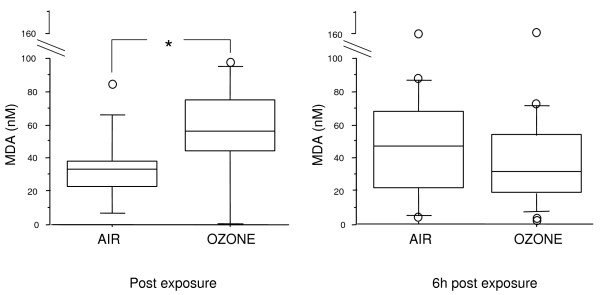
MDA in EBC immediately and 6 hours after ozone/air exposure in all subjects (responders plus nonresponders); *p < 0,05.

**Figure 2:**
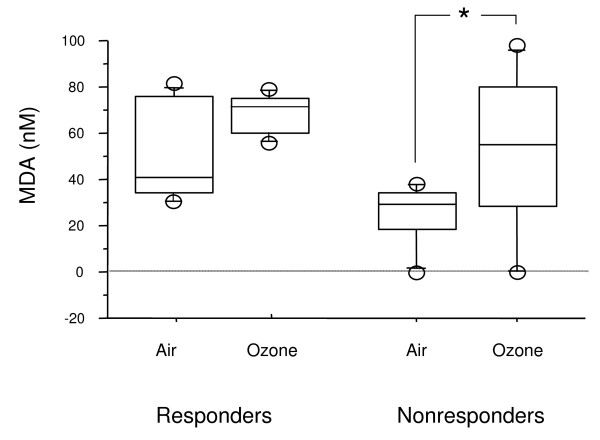
**MDA in EBC immediately after ozone or air exposure in responders and nonresponders**. *p < 0,05.

When all subjects were considered together, MDA levels in EBC were significantly higher immediately after ozone exposure, in comparison with air, but not 6 hours later (Figure 1). When subjects were grouped according to their functional response to ozone, MDA concentrations in EBC increased from baseline in both groups, but the difference was significant only in nonresponders (Figure 2).

Nitric oxide (eNO) levels in exhaled air were above normal value (20 ppb) in 13 out of 23 asthmatic subjects (mean values: 33.7 ± 29.9 ppb and 34.1 ± 29.7 ppb before air and ozone exposure respectively) and did not significantly change immediately and 6 hours after either air or ozone exposures.

No difference in the inflammatory response to ozone exposure was found between subjects bearing *NQO1wt *and *GSTM1null *genotypes and subjects bearing different genotypic combinations.

### Relationship between functional and biological data

**Figure 3:**
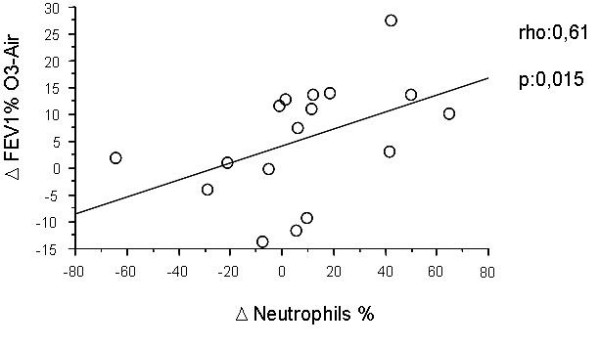
**Correlation between FEV1 fall after ozone (corrected by air) and changes in sputum neutrophils (ΔNeutrophils%) in all subjects (responders plus nonresponders) (Spearman Rank correlation test)**.

Considering all the subjects together, a significant correlation was found between ΔFEV1%_O3-Air _and ozone-air difference sputum neutrophil percentages (Δ N%) (Figure 3). No significant correlation was observed between changes in FEV1 and the ozone-air difference of the other inflammatory markers studied.

No correlation was observed between O3-air changes in MDA immediately after exposure and changes in sputum neutrophils 6 hours after exposure.

After ozone exposure, a positive correlation was found between neutrophil counts (cells/ml) and NE (p = 0.01, rho = 0.6) and IL-8 levels (p = 0.03, rho = 0.6) in induced sputum.

## Discussion

The main result of this study was that some asthmatic subjects defined as "nonresponders" showed however an increase in airway markers of neutrophilic inflammation and oxidative stress, thus suggesting that they are nonetheless sensitive to the effect of ozone. In nonresponders, eosinophils (but not neutrophils) and IL-8 in sputum and MDA in exhaled breath condensate increased after ozone exposure, despite clinically nonsignificant changes in pulmonary function. The inverse relationship between sputum neutrophils and FEV1 values after exposure was significant only when responders and nonresponders were taken all together.

Only a small subgroup of the 23 asthmatic subjects studied had a functional airway response to ozone, which was associated with airway neutrophil recruitment. This fact may be due to regular pharmacologic treatment with inhaled corticosteroids, which may have blunted the severity of restrictive ventilatory impairment in most patients studied. The data reported in literature about functional and inflammatory airway response to ozone in asthmatic subjects are controversial: while some studies showed a greater inflammatory response in asthmatics compared to normal subjects [28,29], these data have not been reproduced by other authors [30], although all the patients studied were free from treatment with inhaled corticosteroids. In previous studies, we demonstrated that asthmatic patients with persistent asthma, regularly treated with inhaled corticosteroids, had a lower functional response to ozone [6], and that short-period pre-treatment with inhaled or oral corticosteroids had some effect on the airway response to ozone [19,20].

In subjects defined as "nonresponders" to ozone, there was however a mild decrease in FEV1, FVC and VC at different time points after ozone exposure and of no clinical relevance; sputum neutrophils, however, did not significantly increase. Despite the lack of a relevant functional and inflammatory response, ozone exposure still induced a significant immediate increase in MDA in exhaled breath condensate, and a later increase in neutrophilic soluble markers in induced sputum in these subjects. This delayed acute inflammatory response to ozone may predispose asthmatic subjects to greater sensitivity to other specific or non specific triggers of acute bronchoconstriction. Indeed, a relationship between peaks of environmental ozone concentrations and asthma symptoms or Peak Expiratory Flow decrease in asthmatics has been reported, with a time lag of 2-3 days [31,32], even in asthmatic subjects on regular pharmacologic treatment [33]. These observations suggest that regularly treated, well-controlled asthmatic subjects are not completely protected from an increase in airway inflammation and oxidative stress, and this may predispose to subsequent asthma deterioration.

The increase in MDA was observed immediately after ozone exposure but not 6 hours later, when neutrophilic recruitment in the airways was observed. Although no significant correlation was found between the immediate increase in MDA and the late increase in sputum neutrophilc after ozone exposure, we may hypothesize that the oxidative stress induced by ozone in the airway epithelium may be one of several mechanisms of recruitment and activation of neutrophils in the airways.

When both responders and nonresponders were considered together, a significant correlation between changes in FEV1 and changes in sputum neutrophils was observed. This relationship has never been previously shown by other authors, and in general no correlations between functional and biochemical changes induced by ozone have been reported [12-14]. However, all studies have been performed in healthy volunteers with high airway reactivity to ozone, whereas our study included asthmatic subjects with very mild functional response to ozone and a blunted ozone-induced airway inflammation, probably due to the long-term regular treatment with inhaled corticosteroids.

The increase in sputum eosinophils in nonresponders found in the present study is an original observation, and requires to be confirmed by further investigations. Other authors have demonstrated an increase in sputum or BAL eosinophils or ECP levels, or in urinary EPX in asthmatic or healthy subjects exposed to ozone, but the relationship with functional response was not reported [34,35]. In a previous experience, we observed that when ozone exposure is performed 24 hours after an allergen challenge that has induced a late response, sputum eosinophil percentages are increased in comparison to the exposure to air, suggesting that ozone may amplify allergen-induced eosinophil airway inflammation [36].

We studied patients with well controlled asthma, after a short-term withdrawal (24 hours) of the regular treatment with ICS, associated with LABA in the majority of them. This short duration of the treatment withdrawal before each exposure allows to avoid a direct effect of ICS and mainly of LABA on the airway response, but it does not exclude a possible interference from the long-term ICS use. It is well known that the effect of ICS on airway cells may persist up to some weeks after the withdrawal. Because a long-term withdrawal of ICS was not possible in our patients, most of them affected by moderate asthma, we preferred to withdraw both ICS and LABA treatment 24 hours before each exposure, in order to minimize and standardize the possible interference of asthma treatment. This strategy has been used previously by other authors [29].

We found no difference between subjects bearing *NQO1wt *e *GSTM1*null genotypic combination and those with other genotypic combinations, as regards both functional and biological response to ozone exposure. The few works published on this topic, mainly evaluating ozone-induced functional changes, are controversial. Our results are in agreement with two studies performed either after acute or chronic exposure to ozone [15,37], which found no difference in lung function between different genotypic combinations. The small number of subjects bearing the *NQO1wt *and *GSTM1null *genotypic combination (n = 6) and the different time points chosen in our study might explain the lack of concordance with other studies reporting an ozone-induced increase in oxidative stress markers in this group of subjects [15,17].

In conclusion, the increase in some markers of airway inflammation and/or oxidative stress can be observed also in asthmatic subjects who do not functionally respond to ozone. The low rate of functional response can be due to the blunting effect of the previous regular pharmacological treatment in this category of well controlled asthmatics.

## Abbreviations

FEV1: Forced Expiratory Volume in one Second; FVC: Forced Vital Capacity; VC: Vital Capacity; PF: Pulmonary Function; PFT Pulmonary Function Test; PD20FEV1 meth: cumulative dose of methacoline producing a 20% decrease in FEV1 from baseline value; ICS: Inhaled Corticosteroids; EBC: Exhaled Breath Condensate; BAL: Bronchoalveolar Lavage; ROS: Reactive Oxigen Species; eNO: exhaled Nitric Oxide; HS: Hypertonic Solution; DTT: Dithiothreitol; PBS: Phosphate Buffer Solution; NE: Neutrophilic Elastase; MDA: Malondialdehyde; IL-8: Interleukin-8; GSTM1: Glutathione-S-Transferase; NQO1: NAD(P)H:quinone Oxidoreductase; ECP: Eosinophil Cationic Protein; EPX: Eosinophil Peroxidase; LABA: Long Acting Beta Agonists.

## Competing interests

PLP has received in the last 5 years funds from AstraZeneca, Abbott, Boehringer Ingelheim, Chiesi Pharmaceutical, GlaxoSmithKline, and MerckSharp&Dohme, Novartis, Nycomed and Valeas for teaching and research activities.

None of the other authors has any competing interest to declare.

## Authors' contributions

PLP and BV designed the study; ADF, EB, BV, FC and LM supervised clinical and functional evaluations; MLB and SC did the laboratory analysis; FLD the statistical analysis. BV, PLP and MLB wrote the manuscript, and all the authors read and approved the final version of the manuscript.
